# Differences in glomerular filtration rate estimated with the new eGFRcr CKD EPI age and sex 2021 vs. the eGFRcr CKD EPI 2009 formula

**DOI:** 10.1515/almed-2022-0052

**Published:** 2022-07-04

**Authors:** Cristian Ríos Campillo, María P. Sanz de Pedro, Sara Aldana Barcelo, María Auxiliadora Bajo Rubio, Antonio Buño Soto, Rubén Gómez Rioja

**Affiliations:** Service of Clinical Biochemistry, Hospital Universitario La Paz, Madrid, Spain; Service of Nephology, Hospital Universitario La Paz, Madrid, Spain

**Keywords:** eGFRcr CKD EPI 2009 formula, eGFRcr CKD EPI AS 2021, glomerular filtration rate (GFR)

To the Editor,

In routine practice, glomerular filtration rate is estimated from serum creatinine to assess kidney function and perform dose adjustment. Recent clinical guidelines recommend the CKD EPI 2009 formula [1, 2], supported by the Spanish Society of Nephrology and the Spanish Society of Laboratory Medicine [3].

One of the variables employed as a correction factor in the estimated glomerular filtration rate (eGFR) formula is race, assuming that creatinine concentrations are higher in black individuals. The term “race” has more sociological than biological connotations, and inclusion in eGFR estimation formulas has been the subject of intense debate [4], [5], [6].

Cystatin C is an alternative marker of glomerular filtration rate that is not influenced by muscle mass. However, cystatin C measurement methods are not as standardized as creatinine, and blood concentrations of cystatin C are influenced by non-renal factors. Nevertheless, when determination of creatinine alone has limitations for eGFR estimation, it is recommended that cystatin C is tested either alone (eGFRcys CDK EPI 2012) or in combination with creatinine (eGFRcr cys CKD EPI 2012) [7].

The CKD EPI group recently developed new eGFR formulas based on creatinine and cystatin C [8], which modifies constants and eliminates the race factor. The group recommends the new formula and supports the use of cystatin C as a marker that is not influenced by muscle mass and improves adjustment when the combined formula is used.

Since these formulas were assessed in a population with a high percentage of "black” individuals, it was advisable to assess the impact on our population. To such purpose, 14,539 enzymatic creatinine results (creatininase method in SRM967-standardized Atellica Solution^®^ CH Analyzer) of patients of the Unit of Nephrology were reviewed. eGFR was recalculated using the new eGFRcr CKD EPI AS 2021 formula. In our context, the correction factor “race” has not been considered for the implementation of the conventional formula (eGFRcr CKD EPI 2009), given that race is determined on the medical history based on a subjective assessment. Kidney function can be assessed using alternative methods, such as 24-h creatinine clearance, or by cystatin C determination.

Comparison of eGFR results showed a constant proportional bias of +1.82% in results of the new eGFRcr CKD EPI AS 2021 formula, with respect to the conventional formula. Thus, it was necessary to reclassify 10.3% of results for the different stages of chronic kidney disease established in KDIGO 2012 guidelines (Table 1). Of note, 17.4% of results for stage G3a (eGFR 45–59 mL/min) patients were reclassified as stage G2 (eGFR 60–89 mL/min), whereas 12.4% of stage G4 (eGFR 15–29 mL/min) patients were reclassified as G3b (eGFR 30–44 mL/min), the widely accepted cut-off points for patient referral to the nephrologist and dose adjustment, respectively.

**Table 1:** Reclassification of results by KDIGO (G1-G5) stage of the eGFRcr CDK EPI 2009 versus eGFRcr CKD EPI 2012 formula.

Change of stage	Number of results of patients by stage using eGFRcr CKD EPI 2009	Number of reclassified results	% of reclassified results
G2 → G1	1,865	187	10.0%
G3a → G2	1,934	335	17.4%
G3b → G3a	2,673	377	14.1%
G4 → G3b	3,117	386	12.4%
G5 → G4	3,819	211	5.5%
Total	14,539	1.497	10.3%

Other studies compared the classification power of this new formula, as compared to glomerular filtration rate measured based on iothalamate clearance, with similar results as in our study [9].

This study has some limitations, primarily the selection bias resulting from only including patients of the Unit of Nephrology. Another limitation is that albuminuria was not considered for CKD staging, and race was not included in the conventional formula. This study is intended to anticipate the results of implementing the new eGFRcr CKD EPI AS 2021 formula in our population.

Further studies are warranted to compare these new formulas against estimated glomerular filtration rate measured in new cohorts of a matched population. Since implementing this new procedure in clinical practice is challenging, each laboratory should assess the impact of the new formulas on its local population. As alternative complementary methods for assessing kidney function, laboratories should also consider other formulas recommended in clinical guidelines [2, 10], as well as other markers such as cystatin C, or 24-urine collection.
